# Associated Factors of Caregiver Burden in Parkinson’s Disease: A Systematic Review and Meta-analysis

**DOI:** 10.1093/geroni/igaf122.2350

**Published:** 2025-12-31

**Authors:** Wilfred Wing Fung Sin, Lily Man Lee Chan, Christine Tsz Ying Ng, Edmond Pui Hang Choi, Kris Yuet Wan Lok, Daniel Yee Tak Fong, Jojo Yan Yan Kwok

**Affiliations:** The University of Hong Kong, Hong Kong, China (People’s Republic); The University of Hong Kong, Hong Kong, China (People’s Republic); The University of Hong Kong, Hong Kong, China (People’s Republic); The University of Hong Kong, Hong Kong, China (People’s Republic); The University of Hong Kong, Hong Kong, China (People’s Republic); The University of Hong Kong, Hong Kong, China (People’s Republic); The University of Hong Kong, Hong Kong, China (People’s Republic)

## Abstract

Informal caregiving for Parkinson’s disease (PD) patients could be challenging and often results in severe caregiver burden. This systematic review and meta-analysis, guided by the Stress-Appraisal Model, aimed to identify factors associated with caregiver burden. A search of five databases from inception to February 2025 identified 66 studies involving 30,533 patients and 30,419 caregivers. Of 106 factors identified (78 patient-level, 28 caregiver-level), 41 were included in random-effects meta-analyses. Patients’ neuropsychiatric symptom severity (r = 0.57, 95% CI = 0.35–0.73, p < 0.001) and caregivers’ psychological distress (r = 0.46, 95% CI = 0.38–0.53, p < 0.001) showed the strongest, albeit moderate, associations with greater caregiver burden. Other factors—including patients’ advanced disease stage, disability, disease duration, motor symptoms, and caregivers’ caregiving hours, social support, and care dyad demographic characteristics—demonstrated weak associations with caregiver burden. Meta-regression indicated stronger correlation coefficients among studies from Southeast Asia and Western Pacific regions These findings highlight the multidimensional nature of caregiver burden of caregiver burden in Parkinson’s disease informal caregivers, primarily associated with patients’ neuropsychiatric symptom severity and caregivers’ psychological distress, with cultural contexts amplifying burden in certain regions. Further research on psychological resilience, A paradigm shift is need to prioritize modifiable traits and protective factors among PD caregiver-patient dyads, and to explore system-level determinants to inform the development culturally adapted interventions for Parkinson’s disease caregiving dyads.

